# Multifunctional Modified Chitosan Biopolymers for Dual Applications in Biomedical and Industrial Field: Synthesis and Evaluation of Thermal, Chemical, Morphological, Structural, In Vitro Drug-Release Rate, Swelling and Metal Uptake Studies

**DOI:** 10.3390/s22093454

**Published:** 2022-04-30

**Authors:** Lalita Chopra, Jasgurpreet Singh Chohan, Shubham Sharma, Mariusz Pelc, Aleksandra Kawala-Sterniuk

**Affiliations:** 1 Environment Chemistry Laboratory, Department of Chemistry (UIS), Chandigarh University, Mohali 140413, India; lalita.chemistry@cumail.in; 2 Mechanical Engineering Department, University Centre for Research and Development, Chandigarh University, Mohali 140413, India; jaskhera@gmail.com; 3 Department of Mechanical Engineering, IK Gujral Punjab Technical University, Main Campus, Kapurthala 144603, India; 4 Faculty of Electrical Engineering, Automatic Control and Informatics, Opole University of Technology, ul. Proszkowska 76, 45-758 Opole, Poland; m.pelc@po.edu.pl; 5 School of Computing of Mathematical Sciences, Old Royal Naval College, University of Greenwich, Park Row, London SE10 9LS, UK

**Keywords:** binary graft copolymers, backbone, physico-chemical analysis, non-Fickian diffusion, adsorption

## Abstract

The hydrogel materials are getting attention from the research due to their multidimensional usage in various fields. Chitosan is one of the most important hydrogels used in this regard. In this paper multifunctional binary graft copolymeric matrices of chitosan with monomer AA and various comonomers AAm and AN were prepared by performing free radical graft copolymerization in the presence of an initiator KPS. The binary grafting can be done at five different molar concentrations of binary comonomers at already optimized concentration of AA, KPS and other reaction conditions such as time, temperature, solvent amount, etc. Various optimum reaction conditions were investigated and presented in this work; the backbone as well as binary grafts Ch-graft-poly (AA-cop-AAm) and Ch-graft-poly (AA-cop-AN) were characterized via various physio-chemical techniques of analysis such as SEM analysis, Xray diffraction (XRD), TGA/DTA and FTIR. In the batch experiments, the binary grafts were investigated for the percent swelling with respect to pH (pH of 2.2, 7.0, 7.4 and 9.4) and time (contact time 1 to 24 h). Uploading and controllable in vitro release of the drug DS (anti-inflammatory) was examined with reverence to gastrointestinal pH and time. The binary grafts showed significantly better-controlled drug diffusion than the unmodified backbone. The kinetic study revealed that the diffusion of the drug occurred by the non-Fickian way. In the case of separation technologies, experiments (batch tests) were executed for the toxic bivalent metal ions Fe (II) and Pb (II) sorption from the aqueous media with respect to the parameters such as interaction period, concentration of fed metal ions in solution, pH and temperature. The binary grafted matrices showed superior results compared to chitosan. The kinetics study revealed that the matrices show pseudo-second order adsorption. The graft copolymer Ch-graft-poly (AA-cop-AAm) provided superior results in sustainable drug release as well as metal ion uptake. The study explored the potential of chitosan-based materials in the industry as well in the biomedical field. The results proved these to be excellent materials with a lot of potential as adsorbents.

## 1. Introduction

Nowadays, the two major scientifically concerned areas are the biomedical industry (alternate and effective resources) and chemical industry waste management, which is leading to the huge production of environmental pollution. This has generated the necessity of environment conservation as one of the economical and social concerns [1]. Today, the utmost pitiless snags concerning pollution is the adulteration of the water resources by the heavy metal ions resulting from the development of the new agricultural practices, mining activities, liquid metal and dye waste discharge from industries such as paper, battery, refineries, metallurgy and textile. The waste discharge containing toxic metal ions may cause health hazards to the living organisms [2,3]. Thus, elimination of these heavy and toxic metal ions is a prime challenge for numerous researchers around the world [4] due to the non-biodegradable, lethal and bio accumulative nature of these, as even traces of these metal ions affect living organisms [5]. For these purposes, numerous approaches were applied in order to exclude toxic metal ions such as electrocoagulation, electro-spun, flocculation [6]. The traditional approaches were however, expensive and less effective; also, the consequence to the metal sludge production has been confirmed to be fairly intricate for elimination [7]. In contrast to the mentioned old-style methods, the adsorption of the metal ions by bio-adsorbents proved to be a better substitute [8]. The use of biopolymers is also advantageous because of their renewable origin, cost-effectiveness, further efficient and hence provides alternative resources. The second major concern was to develop the non-toxic biodegradable matrices for the sustainable and controlled drug release [9]. The target-oriented drug delivery vehicle delivers the drug at a proportion required as per bodily need over a specific time period and also on a specific target. Sustainable drug delivery systems allow controlled and slow release of the drug in the specific cells/tissues/organs [10]. The drug delivery systems work by incorporating the therapeutic agents inside the polymer system in a way that drug is freed at the target in a well-defined and controlled manner [11]. Drug delivery systems were having a limitless combination of their wide-ranging hydrophilic and hydrophobic components and also the interactions such as polymeric–polymeric, polymeric–drug and polymeric–solvent interactions [12]. The polymeric hydrogels have numerous functional groups that make them an admirable applicant for drug delivery vehicles. The inclusion of the drug in the hydrogel matrix having hydrophilic groups for better polymer-drug interaction is a technique of making controlled drug release formulations [13]. The literature background study revealed that biopolymeric matrices (unmodified as well as modified one such as graft copolymers, crosslinked copolymers, IPN, semi-IPN, composites) were applied in the stimulus-targeted or sustainable release of variety of drugs such as diclofenac sodium (DS) Insulin, 5-Fluro uracil (5-FU), nifedipine (NFD), ketoprofen, ibuprofen, berberine, etc. [11,14,15].

Diclofenac sodium (DS) is an anti-inflammatory gastrointestinal drug used in various in vitro and in vivo experiments to study its uptake as well as controlled diffusion by hydrogel-based matrices, beads, gels, capsules, etc. The literature survey for the use of biopolymer-based hydrogel materials for the targeted delivery of DS. Baolong Niu et al. synthesized and characterized hydrogel beads of sodium-loaded sodium alginate/carboxymethyl chitosan—ZnO and experimented for in vivo as well as in -vitro release of DS drug. In vivo studies revealed that the beads proved to be efficient for oral drug administration. The release of the drug is sustainable and slow [16]. With this in vivo pharmacokinetics investigation was performed on rats and the rats administrated with the above said hydrogel beads showed better bioavailability of DS drug as compared to oral administration [16]. Hence, the study proved that SA/CMCS—ZnO hydrogel beads were potential candidates for drug delivery in gastrointestinal environment. Similarly, Nafisa Gull and coworkers in 2020 presented their work on the inflammation responsive chitosan-based hydrogels for DS release. Polyvinyl pyrrolidone and chitosan were utilized as base and crosslinking is done with epichlorohydrin. Hydrogels films were fabricated by employing solution casting technique. Drug encapsulation efficiency was found to be 84% and drug release was more than 87% within 130 min, hence proved excellent candidates for medical procedures [17]. In 2021, Li Shan Tan et al. fabricated crosslinked hydrogels of carboxymethyl sago pulp/chitosan loaded with DS to investigate DS release at different pHs [18]. Very little amount of DS drug, less than 5%, was released slowly at pH 1.2 (stomach pH) by following first order kinetic model of drug diffusion. Antimicrobial properties exhibited by the hydrogels loaded with DS drug was remarkable with against Escherichia coli, Pseudomonas aeruginosa and other bacterial strains [18]. Nature is abundant with many biopolymers such as cellulose, chitin, chitosan, starch, dextrin, Guargum and many more. Among biopolymers, chitosan is of special interest as it is generated from the sea waste material. It is bifunctional polymer with $--NH_{2}$ group in addition to the −OH functionality and these functional groups help the biopolymer into novel binding to the metal ions as well as with drugs. Along with number of unique or green properties such as zero waste, non-toxic nature, bio-compatibility and biodegradable nature, some limitations are there with the efficient utilization such as flaky nature, less solubility, low thermal stability, etc. However, its properties can be amended by graft copolymerization consequence into the betterment of properties such as porosity, swelling due to the assimilation of a number of functional groups [19,20]. Chitosan is a bipolymer obtained from chitin and is non-toxic, biocompatible, bioactive, and biodegradable [20,21,22]. It has numerous applications in various fields such as among the others: agriculture, food industry, medicine, paper fabrication, textile industry, and water treatment; additionally, chitosan has a good film-forming ability widely used for development of sensors and biosensors [21,23,24,25,26]. Numerous scientific reports prove chitin to be one of the most important polymers of the whole 21st century. They are also a building block of diatoms, crustaceans and insects skeletons [23]. Along with the various vital characteristics such as bio-origin, non-toxicity, biocompatibility and others, chitosan exhibits the limitations being too flaky and less porous. Therefore, the needful fabrication can improve its properties [24]. Comprehensive research work is reported on modification of chitosan by graft co-polymerization with different vinyl monomers which improve the size and number of pores, hydrogel character and also the addition of specific functional groups such as carboxylic, amide, nitrile, ester and epoxy functional groups which cause greater chelation to the metal ions [27]. Grafting provides a mode to incorporate some new and desired functional groups onto chitosan so as to enhance horizon of its applications [24,28]. Graft copolymerisation of binary comonomers along with the monomer on the chitosan backbone will widen its spectrum of applications due to selective addition of new functional groups onto it and strength of the polymer also increases [29]. In this paper at the already optimised reaction conditions evaluated for AA, binary vinyl comonomer mixtures acrylamide and acrylonitrile were graft copolymerized onto chitosan by using five changed molar concentrations of comonomers with respect to AA. Optimum concentration of acrylamide and acrylonitrile were obtained by using five different molar concentrations of AAm and AN. Chitosan and binary grafted copolymers were analysed for the change in morphological, structural and thermal properties by characterization techniques such as SEM, XRD, FTIR, TGA/DTA. Chitosan and synthesized binary grafts were investigated for the swelling study at the different pH 2.2, 6.8, 7.0, 7.4 and 9.4 in order to find out their applications in metal ion sorption and controlled drug delivery. The synthesized matrices were used as bio-adsorbent materials for the removal of bivalent metal ions Fe(II) and Pb(II) from the aqueous medium at different pH, contact time, temperature and metal ion feed concentration. The kinetic studies were performed by using pseudo-first and second-order kinetic models. The matrices were also investigated for the uptake of DS drug at room temperature and pH 7.0. The drug release by the matrices were investigated at different stimuli in order to check optimum conditions for the sustainable release of gastrointestinal drug DS.

## 2. Materials and Methods

For this study purposes 75–85% deacetylated chitosan (LMW chitosan of 50,000–190,000 Da) from Sisco Research Laboratories Private Ltd., KPS (from Ranbaxy, SAS Nagar Mohali, India), DS (Sigma Aldrich, Saint Louis, MO, USA), acrylic acid (from Merck), acrylamide (from Merck), Acrylonitrile (from SD Fine), were applied as customary without any further purification. The authors also used DS (Sigma Aldrich), HCl, NaOH, KH${}_{2}$PO${}_{4}$, KCl and Na${}_{2}B_{4}O_{7}$. In addition $10H_{2}O$ (SD Fine, India) has been used for preparing buffer solutions of different pH. Ferrous sulfate ($FeSO_{4}$), Lead nitrate ($Pb{\left(NO_{3}\right)}_{2}$) (SD Fine) were used for making solutions of particular metal ion in deionized water.

### 2.1. Production of Binary Grafted Copolymers

At the already optimised graft copolymerization reaction settings of AA alone onto chitosan [30], two comonomers Acrylamide (AAm) and Acrylonitrile AN (binary grafting) were graft copolymerized onto chitosan along with AA at five different molar concentrations of comonomers with respect to the AA in terms of increasing molar ratio. In the product polymeric gels mixture, the homopolymer formed along with copolymers were removed by the already documented solvent extraction process [30,31]. The reaction products formed were stirred vigorously in the solvent mixtures which solubilize homopolymers. The extraction practice was continued until a static weight of the product was achieved. The solvent mixture used were methanol-water for poly (AA-cop-AAm), poly (AAm) and poly (AA), methanol-DMF for poly (AA-cop-AN), poly (AN) and poly (AA) removal. Repeated the extraction continued until the constant weight of binary grafting was obtained after drying them at room temperature. The kinetics of the binary graft copolymerization was studied in terms of percent graft copolymerization ($P_{g}$) and percent graft copolymerization efficiency (%GE) for the binary graft copolymeric matrices were studied. The $P_{g}$ and %GE were calculated and expressed withe following Equations (1) and (2):

(1) $$P_{g}=\frac{P_{1}-P_{0}}{P_{0}}\times 100,$$

(2) $$\%GE=\frac{P_{1}-P_{0}}{M_{1}}\times 100,$$

where:

- $P_{0}$ is the backbone weight,
- $P_{1}$ is the product’s weight (binary grafts synthesized),
- $M_{1}$ is the sum of the monomer’s weights.

### 2.2. Metal Ion Uptake

Metal ion adsorption study was performed for bio-sorbents chitosan and binary matrices by equilibration method. The standard solutions of metal ions having concentration 5.00 mg/L for the respective metal ions were made by the dissolution of calculated weights of $FeSO_{4}$, $7H_{2}O$ and $Pb{\left(NO_{3}\right)}_{2}$ in deionized water. For the sorption studies 250 mg of polymer samples were dipped in 10.00 mL metal ion solution (only single metal ion) at particular contact time, pH, temperature and fed metal ion concentration one by one to evaluate the optimum conditions of contact time, temperature, pH and metal ion feed concentration. The solution was observed for the rejected ions concentration on DR 3900 Spectrophotometer (HACH Co., Loveland, CO, USA) by using standard pillow reagents. Parameter contact time, pH, temperature and individually fed metal ion concentration were varied one by one in order to evaluate optimum sorption conditions [32]. Metal ion sorption parameters such as retention capacity ($Q_{r}$), percent uptake ($P_{u}$) as well as partition coefficient ($K_{d}$) were calculated as follows ((3)–(5)):

(3) $$P_{u}=\frac{C_{o}-P_{r}}{C_{o}}\times 100,$$

(4) $$K_{d}=\frac{C_{a}}{C_{r}}\times \frac{V(mL)}{W_{p}\left(g\right)},$$

(5) $$Q_{r}=\frac{C_{a}\left(mEQ\right)}{W_{p}\left(g\right)},$$

where:

- $C_{o}$ is fed metal ions concentration,
- $C_{r}$ is rejected metal ions concentration,
- $C_{a}$ is metal ions concentration ion in polymeric matrix,
- *V* is solution volume in mL,
- $W_{p}$ is dry polymeric weight in grams.

### 2.3. Kinetic Models

Metal ion adsorption was kinetically modelled by using pseudo-first-order as well as pseudo-second-order kinetic models. The Metal ions uptake’s kinetic studies provide statistics regarding bio-adsorption mechanism and the efficiency of the bio-adsorbent for a specific metal ion. Various kinetic models were applied on the kinetics study of metal ion sorption by the polymeric matrices such as Langmuir, Freundlich, etc. Here in this research article, we had applied two models—pseudo-first-order (6) and second-order (7) kinetic models [33]:

(6) $$log\left(q_{e}-q_{t}\right)=logq_{e}-\frac{k_{1}}{2.303}t,$$

where:

- $k_{1}$—equilibrium rate constant for pseudo-first-order model ($T^{-1}$);
- $q_{e}$ and $q_{t}$—sorption measurements in mg/g at equilibrium period and at time *t*, respectively.

(7) $$\frac{t}{q_{t}}=\frac{t}{q_{e}}+\frac{1}{k_{2}q_{e}^{2}},$$

where:

- $k_{2}$—equilibrium rate constant for pseudo-second-order kinetic model (g $mg^{-1}$$T^{-1}$);
- $q_{e}$ and $q_{t}$—sorption measurements in mg/g at equilibrium period and at time *t*, respectively.

In the case of pseudo-first-order kinetic models, a curve is plotted between $log(q_{e}-q_{t}$) vs $t,q_{e}$ and $k_{1}$ are obtained from plot’s intercept and slope, respectively, of the straight-line graph plotted. If straight line graph showed that metal ion uptake follows pseudo-first-order kinetic mechanism but nonlinearity of graph showed that pseudo-first-order kinetic reaction model is incompetent. For pseudo-second-order kinetic model, the metal ion adsorption rate is directly dependent upon the quantity of vacant sites square. Values of $q_{e}$ and $k_{2}$ obtained from the plot’s slope and intercept, respectively, when $\frac{t}{q_{t}}$ plotted against *t*. The literature survey revealed that the pseudo-second-order kinetic model is more valuable than the pseudo-first-order model because the first one illustrates a steady alteration in rate constant values over a considerable range of initial concentration of metal ions.

### 2.4. Diclofenac Sodium Drug Uptake and Release Studies

**Figure 1:**
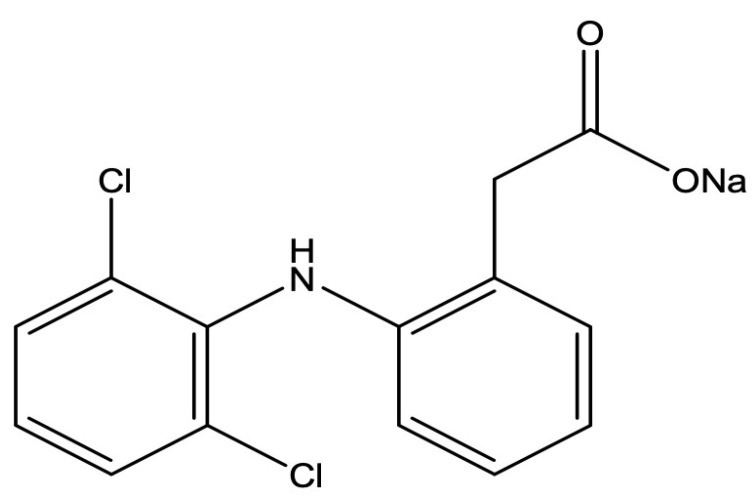
Diclofenac sodium (Sodium salt of 2-(2, 6-dichloroaniline) phenylacetic acid).

Model drug Diclofenac Sodium (DS) is a gastrointestinal drug having long-lasting analgesic and antipyretic properties (see Figure 1). The DS is considered to be an ideal candidate for the study of controlled or sustainable drug release because of its very petite half-life and related hostile effects. The DS can solubilize in the intestinal fluid as well as in water [34].

In order to study the uptake and release of model drug DS, 100 μg/mL stock solution of drug was prepared by taking all the necessary precautions. The solution was scanned for absorption at 276 nm (based on thorough literature background) wavelength by using double beam UV-Visible spectrophotometer. From these absorption values a standard graph was plotted for absorption v/s drug concentration (drug concentration varied 2.00 μg/mL to 100.00 μg/mL. In order to upload the DS drug into candidate polymers, 25.00 mg polymeric sample was weighed and dipped for 24 h in 10.00 mL drug solution of 100 μg/mL DS concentration. After 24 h, the solution was analyzed for the DS concentration left in the mother liquid after adsorption. Amount of drug uptake by the polymeric sample was calculated by deducting left DS concentration in the filtrate from the original concentration of the DS taken. The drug loaded tasters were dried out for 3–4 days in a room temperature.

The drug release through bare chitosan and selected grafts was investigated by submerging the polymeric samples in solutions of different pH 2.2, 7.0, 7.4 and 9.4. Percent drug released by the matrices was studied with respect to time after 1,2,3,4,5,6 and 24 h. Buffer solutions of different pH were fabricated by using KCl, HCl, KH${}_{2}PO_{4}$, NaOH and $Na_{2}B_{4}O_{7}$. The $10H_{2}O$ (borax) solutions of specific volumes and molarity. Drug uptake and percent drug releases by the polymeric samples with respect to time were calculated can be expressed as below ((8) and (9)):

(8) $$P_{du}=\frac{D_{o}-D_{l}}{D_{o}}\times 100,$$

(9) $$P_{dr}=\frac{D_{s}}{D_{o}}\times 100,$$

where:

- $P_{du}$—percent drug uptake,
- $D_{o}$—total drug taken in solution,
- $D_{l}$—drug left in supernatant liquid,
- $P_{dr}$—percent drug release,
- $D_{s}$—concentration of drug in solution.

### 2.5. Drug Release Kinetics

Fick’s Law/Korsmeyer–Peppas equation serves to understand drug diffusion mechanisms describing the relationship between drug diffusion through a polymeric sample with respect to time. Diffusion of the drug from the polymeric samples can be Fickian or non-Fickian and anomalous. In the case of Fickian diffusion, the solute transport takes place with polymer relaxation [35] time ($t_{r}$) sample larger as compared to the solvent diffusion time ($t_{d}$) non-Fickian $t_{r}\approx t_{d}$. According to the Fick’s law, the fraction of drug release at a particular time t was specified by the following Equation (10):

(10) $$F=\frac{M_{t}}{M_{\infty }}=kt^{n}.$$

Taking log on both the sides (11):

(11) $$log\frac{M_{t}}{M_{\infty }}=logk+nlogt,$$

where:

- *F*—fraction of drug released at contact time *t*, $M_{t}$—the amount of drug released at time *t*,
- $M_{\infty }$—drug released at infinite time,
- *n*—diffusion exponent,
- *k*—gel characteristics constant,
- *t*—time in hours.

The *n* value found from the slope of linear regression graph between $log\frac{M_{t}}{M_{\infty }}$ verses log *t*, whereas *k* has been assessed through the plot’s intercept. The correlation coefficient *r* value if approaching to the unity signifies linear drug diffusion by matrices. If *n* is lower than 0.5, then it indicates that the release of the drug from the polymeric sample took place by Fickian diffusion. If n=0.5, the drug release or diffusion through the matrices follows the unidirectional or Fickian drug diffusion. In the case of n>0.5, drug diffusion ensues by anomalous mode or non-Fickian mode. For n=1, release took place by wholly non-Fickian way or follows Case II release. If *n* lies intermediate between 0.5 and 1.0 diffusive transport credited to the inconsistent/anomalous release and if n>1.0, it offers non-Fickian super case—II kind of diffusion.

## 3. Results and Discussion

In the further part of this work both results and discussion of this study have been in detail presented.

### 3.1. Optimization of Comonomer Concentration

In this reaction mechanism, comonomers AAm and AN were grafted along with the AA onto chitosan at already optimized reaction conditions evaluated for AA alone [solvent ($H_{2}O)=20$ mL, $\left[KPS\right]=7.5\times 10^{-2}$ moles/L, $\left[AA\right]=109.39\times 10^{-2}$ moles/L, time of reaction = 1 h, temperature of reaction = 70 °C] [30]. Comonomer’s molar concentration was varied in molar ration of 0.5,1.0,1.5,2.0 and 2.5 at the optimum molar concentration of AA.

**Figure 2:**
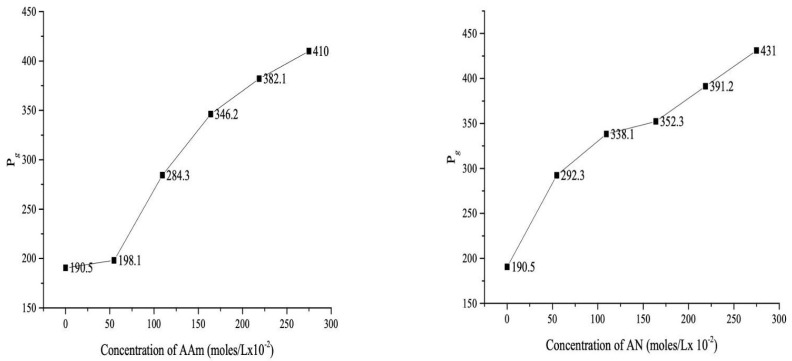
Effect of concentration of AAm and AN: amount of $H_{2}O=20$ mL, $\left[AA\right]=109.39\times 10^{-2}$ moles/L, $\left[KPS\right]=7.5\times 10^{-2}$ moles/L, reaction time = 1 h, reaction temperature = 70 °C.

The AAm and AN concentrations were changed from $59.69\times 10^{-2}$ moles/L to $274.97\times 10^{-2}$ moles/L with constant concentration of $AA=109.39\times 10^{-2}$ moles/L. With increase in comonomer AAm and AN) concentration the $P_{g}$ increases gradually and maximum $P_{g}$ of 410.00 was also recorded at $247.97\times 10^{-2}$ moles/L AAm concentration. In addition, the maximum $P_{g}$ recorded as 431.00 at $247.97\times 10^{-2}$ moles/L concentration of AN. The pattern of %GE follow different order as it firstly increases with increase in molar concentration but afterwards it decreases gradually with further rise in concentration of comonomer. The optimum %GE(90.73) at $109.39\times 10^{-2}$ moles/L molar concentration of AAm, whereas in the case of AN, maximum %GE as 151.54 was obtained at $218.78\times 10^{-2}$ moles/L concentration of AN (Figure 2).

Physio-chemical investigation methods such as XRD, SEM, TGA/DTA, FTIR and swelling observations were used for the characterization of the backbone and binary grafts. These techniques were applied in order to get information of variations in surface morphology, incorporation of the comonomer’s and monomer’s functional groups, crystallinity, thermal stability and swelling characteristics, respectively.

### 3.2. Analysis of Binary Grafted Copolymers

The above mentioned methods are also applied for carrying out the elemental, structural, and morphological analyses of biosensors [36].

#### 3.2.1. SEM Study

**Figure 3:**
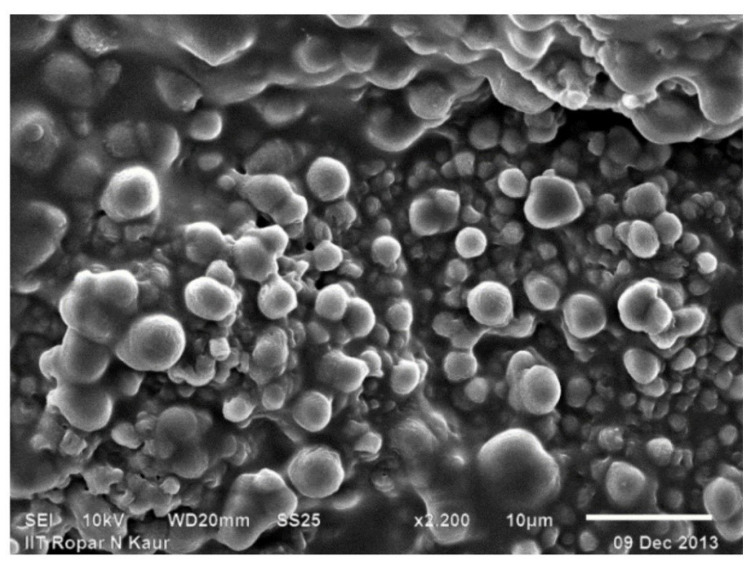
SEM photograph of Ch-graft-poly (AA-cop-AAm).

**Figure 4:**
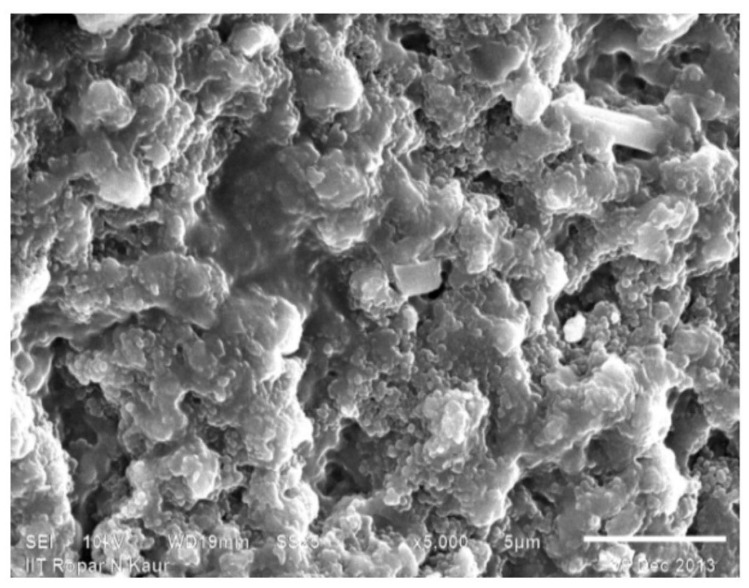
SEM photograph of Ch-graft-poly (AA-cop-AN).

The SEM photographs of chitosan revealed that its surface was composed of abundant folds and was non-particulate, also the particle size is very small and its surface have scarcer pores. The surface was flaky and rough too [30]. The SEM pictures of the binary grafts revealed that there was an extreme alteration in the surface morphology with respect to significant increase in porosity and sponginess, confirming the modification of pristine chitosan by binary graft copolymerization (Figure 3 and Figure 4).

#### 3.2.2. FTIR Study

**Figure 5:**
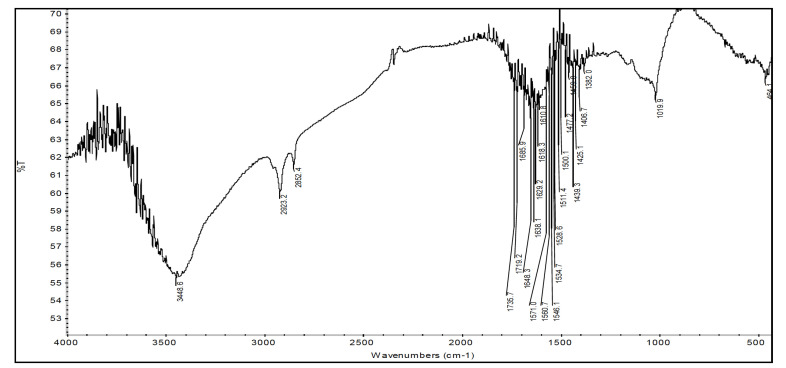
FTIR of Ch-graft-poly (AA-cop-AAm).

**Figure 6:**
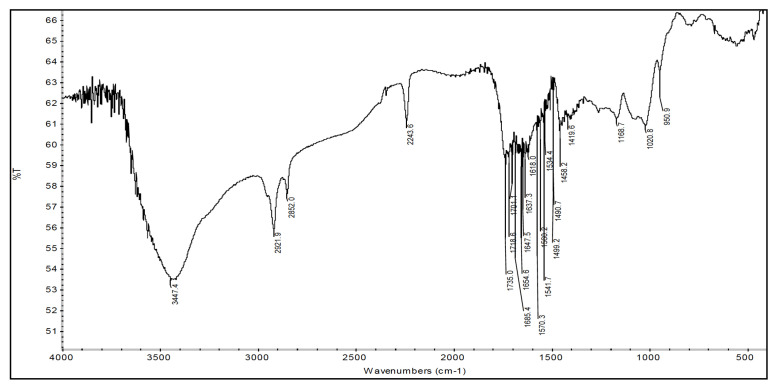
FTIR of Ch-graft-poly (AA-cop-AN).

The FTIR of bare chitosan explained in earlier communications distinctively showed peaks representing occurrence of alcohol, among the others: C-O, N-H and C-N, and characteristic peaks of chitosan moieties [37], whereas the FTIRs of binary grafts when compared with chitosan’s FTIR showed the presence of additional peaks along with the diminishing of some old one. The FTIR spectrum of binary graft copolymer Ch-graft-poly (AA-cop-AAm) displayed peaks at 1685.9 cm${}^{-1}$ attributed to stretching vibrations of -COOH group and at 1719.2 and owing to stretching of >C=O of the amide group. At 1406.7 cm${}^{-1}$ peak obtained because of coupled vibrations of stretching of C-O with in-plane bending of O–H group (this is a feature of -COOH group—see: Figure 5). A particular peak for -COOH group is detected at 1019 cm${}^{-1}$ representing the in-plane O–H group bending. Peaks were obtained at 1648.3 cm${}^{-1}$ (>C=O stretching) and 1618.3 cm${}^{-1}$ (in-plane bending of N-H) for two typical signals for the functional group’s amide-I and amide-II confirming the presence of amide group in the binary graft. The FTIR spectrum of binary graft copolymer Ch-graft-poly (AA-cop-AN) exhibit typical signal of C≡N stretching at 2243.6 cm${}^{-1}$ and recognized the attendance of AN monomer along with the characteristic signals of -COOH group of acrylic acid (Figure 6).

#### 3.2.3. XRD Studies

**Figure 7:**
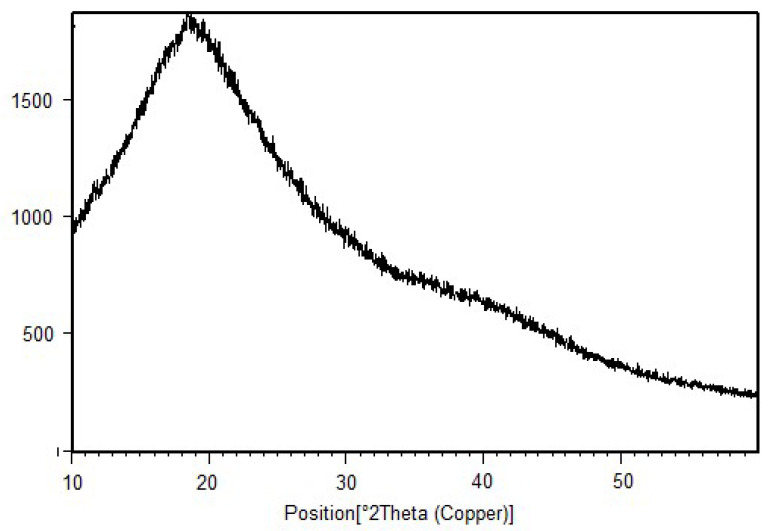
XRD of Ch-graft-poly (AA-cop-AAm).

**Figure 8:**
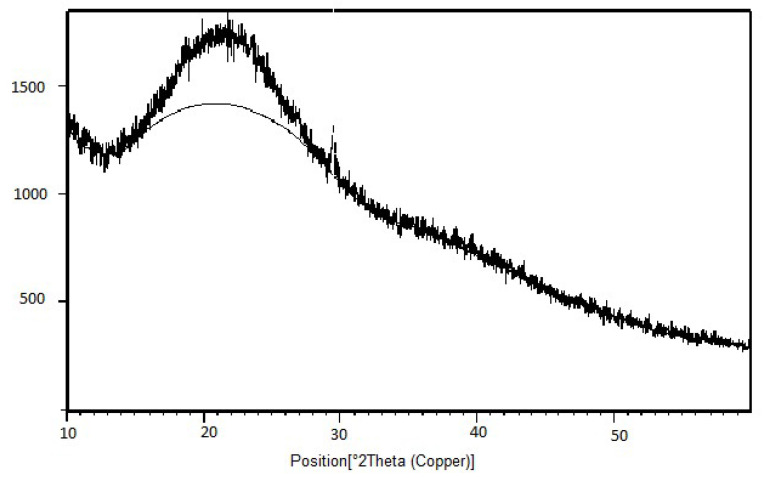
XRD of Ch-graft-poly (AA-cop-AN).

The XRD of the samples were recorded at 2θ=5−−8° using step size 0.0170°. Pristine chitosan XRD already discussed displayed peaks at 10 and at 20 at 2θ positions with merely high comparative intensities representing crystallinity [38]. Compared to chitosan, in the XRD image of the binary graft copolymers there was no sharp peak representing that graft copolymerization crystalline character decreases and amorphous character increases. The loss in peak sharpness after grafting provided the shreds of evidence of grafting (Figure 7 and Figure 8).

#### 3.2.4. Thermal (TGA/DTA) Analysis

**Figure 9:**
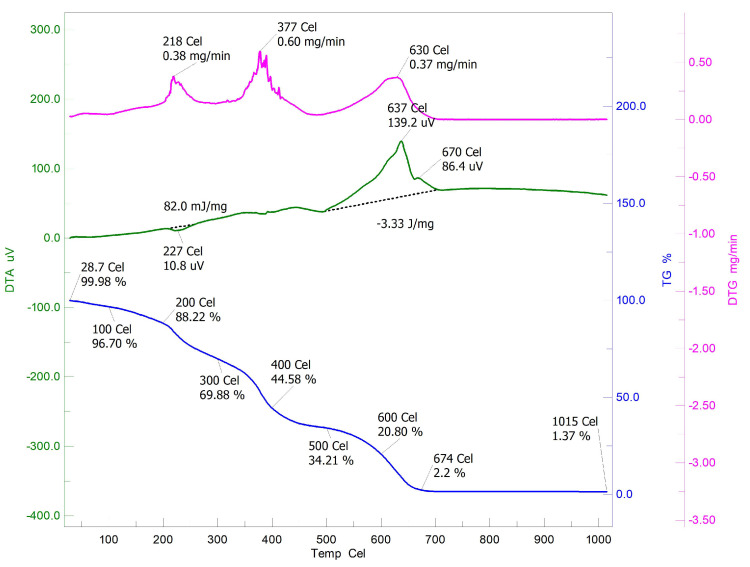
TGA/DTA of Ch-graft-poly (AA-cop-AAm).

The TGA/DTA thermogram of pure chitosan discussed in the earlier part of this paper showed a single stage degradation [30]. Investigation on Ch-graft-poly (AA-cop-AAm) thermograph proved that the graft copolymerization resulted in the enhancement of thermal properties of chitosan as increase in FDT up to 674 °C occurred. There was a three-stage thermal decomposition with maximum weight loss of 79.20% which take place in 300–600 °C. At 674 °C FDP only 2.2% residue remained. In addition, the DTG provided three exothermic peaks around 218 °C, 377 °C and 630 °C for Ch-graft-poly (AA-cop-AAm) which may be due to the decomposition of -COOH and -$CONH_{2}$ groups (Figure 9).

**Figure 10:**
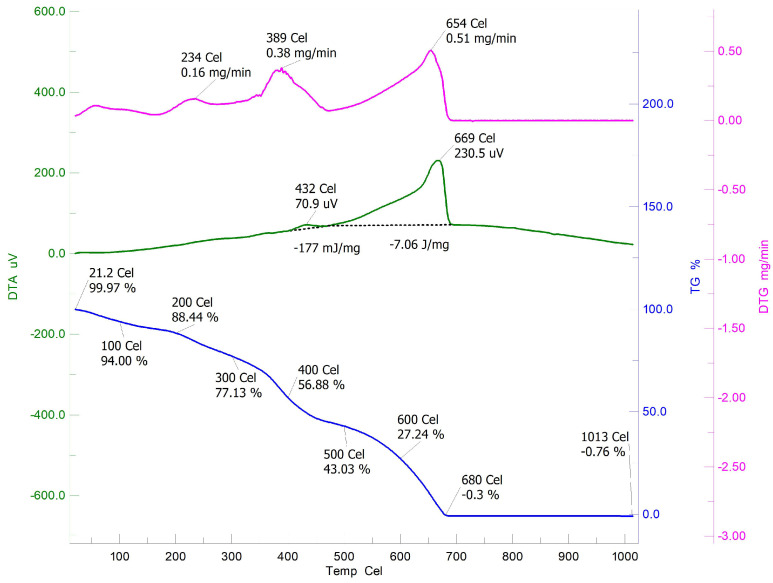
TGA/DTA of Ch-graft-poly (AA-cop-AN).

The TGA of Ch-graft-poly (AA-cop-AN) likewise showed the FDT increased to 680 °C, which is higher as compared to bare chitosan representing increase in thermal stability. Extreme loss in weight occurred in the range of 300–400 °C and only 0.3% residue was left at the FDP. In the DTG three exothermic peaks were observed at 234 °C, 389 °C and 654 °C credited to the degradation of $-CONH_{2}$ and -CN. Analysis the thermograms approved the enhancement of thermal stability of chitosan on binary graft copolymerization (Figure 10).

In Table 1 results of thermogravimetric analyses was presented.

#### 3.2.5. Swelling Study

**Figure 11:**
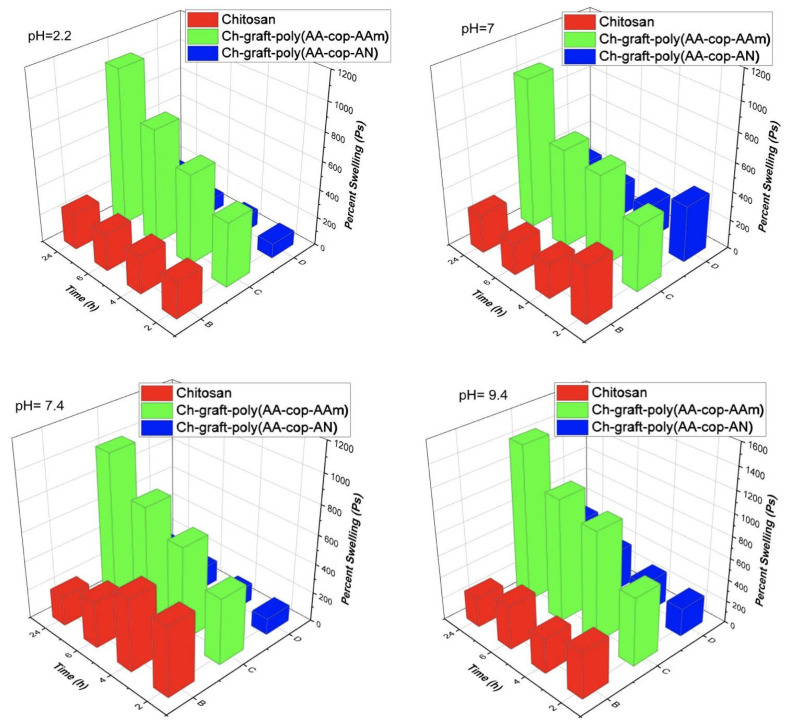
Comparative swelling investigations.

Swelling investigations of bare chitosan and binary graft copolymer were carried out with respect to time at different pH (2.2,7.0,7.4 and 9.4). The $P_{s}$ of chitosan represents very less percent swelling and showed that chitosan swells almost same and very less at all the pH [39]. The binary grafts were more swollen in comparison to pristine chitosan. The Ch-graft-poly (AA-cop-AAm) presented maximum $P_{s}$ of 1096,1020,1012 and 1428 in 24 h at pH 2.2,7.0,7.4 and 9.4, respectively. The $P_{s}$ observed for Ch-graft-poly (AA-cop-AN) was also lesser as compared to Ch-graft-poly (AA-cop-Aam). It showed minimum $P_{s}$ of 172 and 168 in 24 h at pH 2.2 and 7.4, respectively, and the highest $P_{s}$ of 524 in 24 h at 9.4, respectively. Comparative analysis of pristine chitosan and its grafts at various pH with respect to time represented that chitosan as well as binary grafted copolymers swelled to their maximum in strongly alkaline pH 9.4, also chitosan swelled to its maximum in 2 h whereas the grafted binaries keep on swelling and maximum swelling was there in 24 h contact time as presented in Figure 11. The $P_{s}$ (percent swelling) was calculated with (12):

(12) $$P_{s}=\frac{W_{s}-W_{d}}{W_{d}}\times 100,$$

where:

- $P_{s}$—percent swelling of the polymer,
- $W_{s}$—swollen polymer’s weight,
- $W_{d}$—dry polymer’s weight.

#### 3.2.6. Metal Ions Sorption Studies

**Figure 12:**
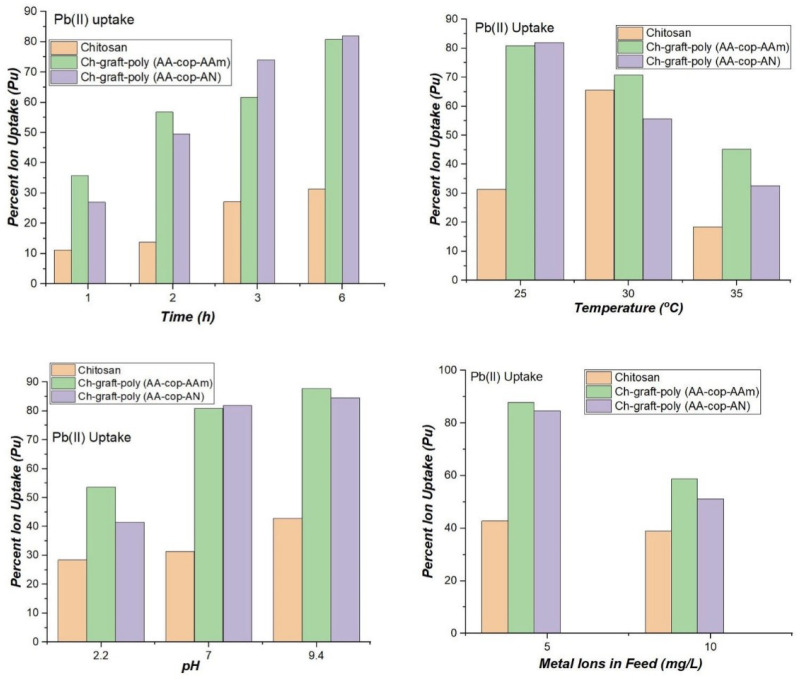
Comparative Pb(II) ions sorption at variable parameters.

In order to study sorption of Pb (II) ions, 250 mg of dried sample was immersed in 10.00 mL metal ion solution comprising of 5.00 mg/L Pb (II) ions at 25 °C and 7.0 pH. The time varied from 1–6 h and with an increase in time percent metal ion uptake also increased. Maximum $P_{u}$ of 82.00% and 80.80% were given by Ch-graft-poly (AA-cop-AN) and Ch-graft-poly (AA-cop-AAm), respectively, which was very high, although, then only 31.40% uptake showed by pristine chitosan. With an increase in temperature at 30 °C and then 35 °C, the percent uptake of Pb(II) ions falls with rising in temperature above 25 °C. This can be attributed to inflamed desorption rate at a high temperature. To investigate the effect of pH, the sorption of Pb(II) ions was studied at pH 2.2 and 9.4 at optimal 6.0 contact hours and 25 °C temperature. Percent uptake showed decrease at pH 2.2 whereas at pH 9.4 an upsurge in $P_{u}$ was noted for the grafted samples. The Ch-graft-poly (AA-cop-AAm) showed highest $P_{u}$ of 87.80. The behaviour showed high resemblance to swelling behaviour of the applicant polymers at pH 2.2 and 9.4. At pH 9.4 swelling of polymers increased because of the formation of sodium salt by acrylic acid and hydrolysis of AAm and AN (Figure 12).

More swelling resulted in more metal ion uptake. Then metal ion feed concentration was enlarged to 10.00 mg/L resulted into a reduction in $P_{u}$ and $K_{d}$ whereas retention capacity of grafted polymers was enhanced.

**Figure 13:**
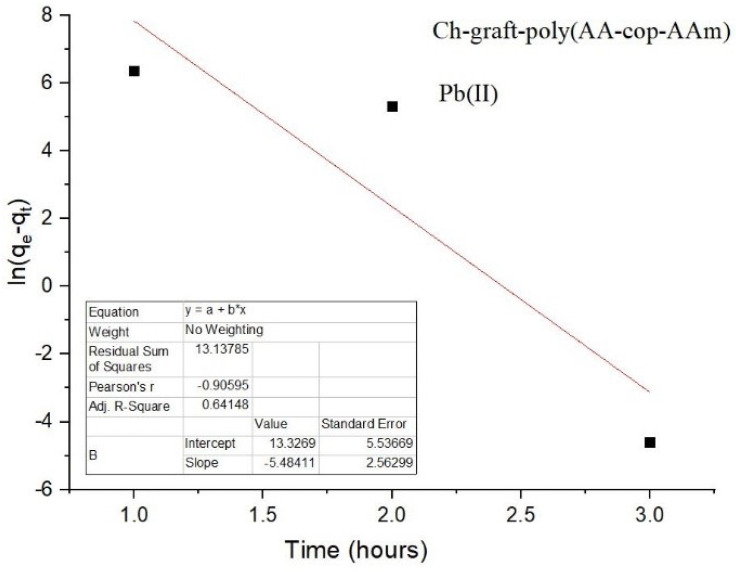
Pseudo-first-order kinetics of Ch-g-poly (AA-co-AAm).

**Figure 14:**
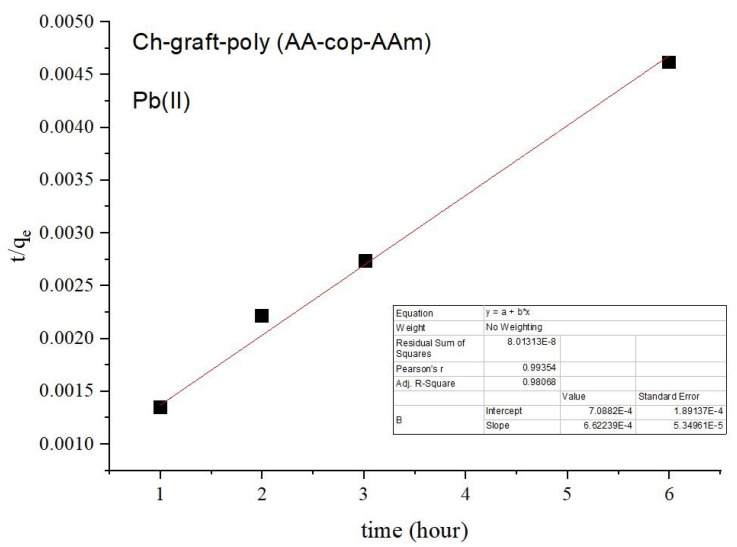
Pseudo-second-order kinetics of Ch-g-poly (AA-co-AAm).

Pseudo-first and pseudo-second-order kinetic models were applied on the adsorption of Pb(II) ions. In the case of the pseudo-second-order model, a straight line is attained with a high degree of linearity. A unitary value of correlation coefficient was observed for this model in disparity to pseudo-first-order kinetic model, as pictured in Figure 13 and Figure 14).

**Figure 15:**
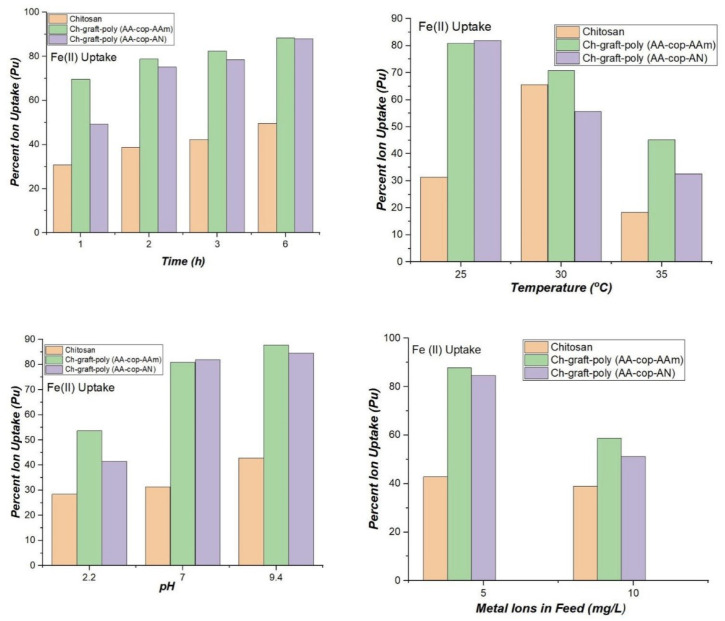
Fe(II) ions sorption at variable parameters.

For the Fe(II) ions sorption, the samples were dipped in 5.00 mg/L solution Fe(II) metal ions concentration at 25 °C temperature and pH 7.0. Altogether tasters displayed a constant upsurge in $P_{u}$, $K_{d}$ and $Q_{r}$ with contact time from 1–6 h. The Ch-graft–poly (AA-cop-AAm) and the Ch-graft-poly (AA-cop-AN) exhibited 88.40% and 88.00% adsorption of Fe(II) from aqueous medium as compared to only 49.60% uptake shown by chitosan. At 6 h contact time, with surge in temperature from 30 °C and 35 °C, all the matrices presented a notable reduction in $P_{}$ apart from bare chitosan showing an increase in $P_{u}$ to 72.00% at 30 °C. When pH was varied to 2.2 at 6 h contact time and optimum temperature 25 °C, a decrease in metal ions uptake was shown by the graft copolymeric samples. Adsorption was not executed at pH 9.4, because of precipitation of Fe(II) ions alkaline medium (Figure 15).

A diminution in $P_{u}$ values for all the matrices were recorded with rise in the Fe(II) ions concentration to 10.00 mg/L at 6 hrs time, 25° and pH 7.0. However, there was an increase in retention capacity for the polymeric samples that may be due of the overall increased Fe(II) ions uptake in weight via weight among the polymeric matrices. The metal ion sorption depends upon the number of aspects such as swelling, porosity, nature as well as the number of functional groups availability, hydrophilicity, monomer’s and comonomer’s nature, the extent of graft copolymerization, crosslinking, etc. The incorporated functional groups showed sorption of metal ions by chelation or by polymer-analogous reactions because of the opening up of polymeric matrix and pores due to swelling. The swelling was enhanced by graft copolymerization of different monomers with hydrophilic functionalities. The opening of the polymeric matrix after swelling resulted in the improved availability of hydrophilic functional groups to interact with positively charged metal ions that imparts a significant role in the amount and choice of metal ion uptake. Apart from all these factors the metal ion’s ionic potential, charge on the ion, size of metal ion, hydration sphere of these metal ions also determines the sorption and retention of metal ions on the polymeric adsorbents.

**Figure 16:**
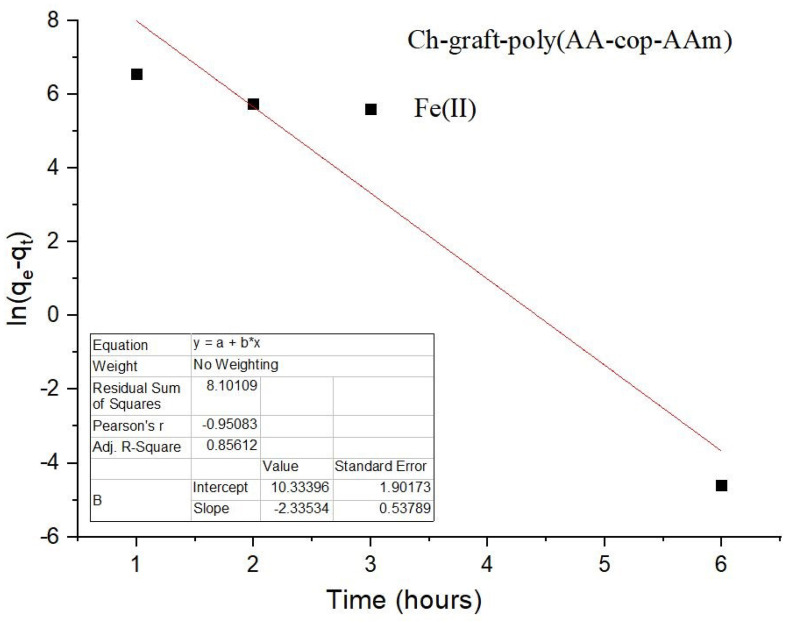
Pseudo-first order kinetics of Ch-graft-poly (AA-cop-AAm).

**Figure 17:**
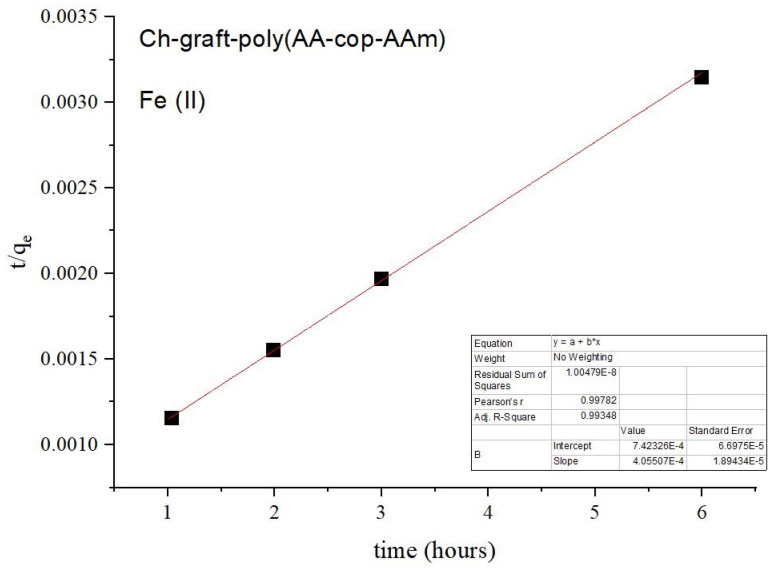
Pseudo-second order kinetics of Ch-graft-poly (AA-cop-AAm).

Kinetic models, pseudo-first and second order were investigated during this study for the purpose of the sorption of Fe(II) ions and a straight line is attained with a high degree of linearity along with unitary value of correlation coefficient in the case of the pseudo-second-order kinetic model, as illustrated with Figure 16 and Figure 17.

#### 3.2.7. Drug Release Behavior and Kinetic Model

The DS was loaded in to the hydrogel samples. In that case the Ch-graft-poly (AA-cop-AAm) showed 42.41% of drug uptake whereas the Ch-graft-poly (AA-cop-AN) exhibited only 5.56% drug uptake which is even less than pristine chitosan (see: Table 2).

**Figure 18:**
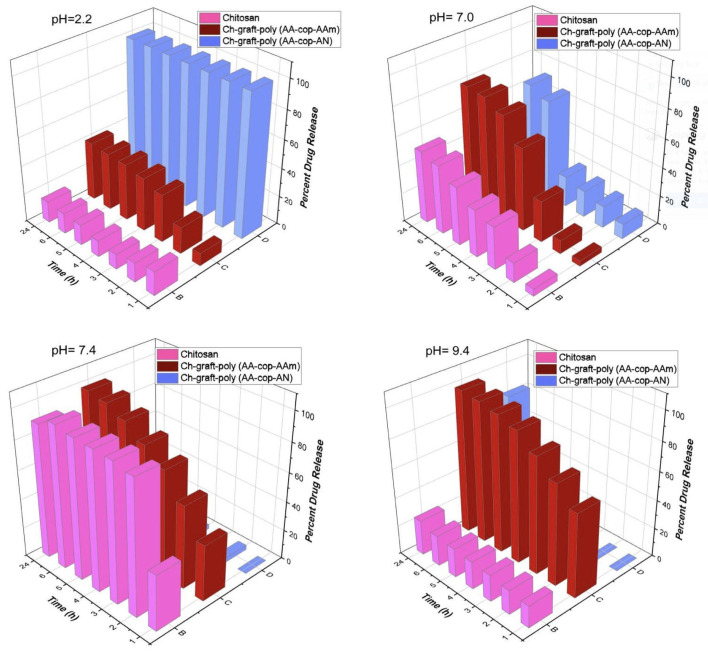
Competitive drug release study.

From drug loaded samples, the drug release study was examined with respect to time and pH. Cumulative release of the DS was very less at acidic pH 2.2 even after 24 h time. Maximum drug release of 40.30% of DS was observed in the case of Ch-graft-poly (AA-cop-AAm) in 6 h. The Ch-graft-poly (AA-cop-AN) released 100% of the drug in 1 h. At 7.0 pH, the rate of drug release became comparatively fast and the highest percent release was given by Ch-graft-poly (AA-cop-AAm) with percent release of 79.09% in 6 h. The percent drug release was more controlled and quite sustainable at pH 7.4 and 9.4 with Ch-graft-poly (AA-cop-AAm) giving the best results for percent release, such as f.e. 96.87% and 97.65% in 6 h at pH 7.4 and 9.4, respectively. At pH 9.4, percent release of the Ch-graft-poly (AA-cop-AN) showed 00.00% drug release in the first 4 h but after that immediately releases all the drug content with the 82.99% drug release in 24 h at pH 9.4. Therefore, the percent drug release rate with respect to pH was observed as 2.2<7.0<7.4<9.4 (see: Figure 18).

The polymeric samples swelled to the different extent at different pH, hence drug molecules diffused into (drug uptake) and diffused out of (drug release) polymeric samples, which allowed the possible use of dry or swollen polymeric samples as drug delivery vehicles. The results obtained showed that prolonged release of DS took place in alkaline media rather than neutral and acidic, which was again related to the swelling behavior of the candidates. Hence, swelling with respect to pH as well as time will decide sustainability as well as targeted drug delivery.

**Figure 19:**
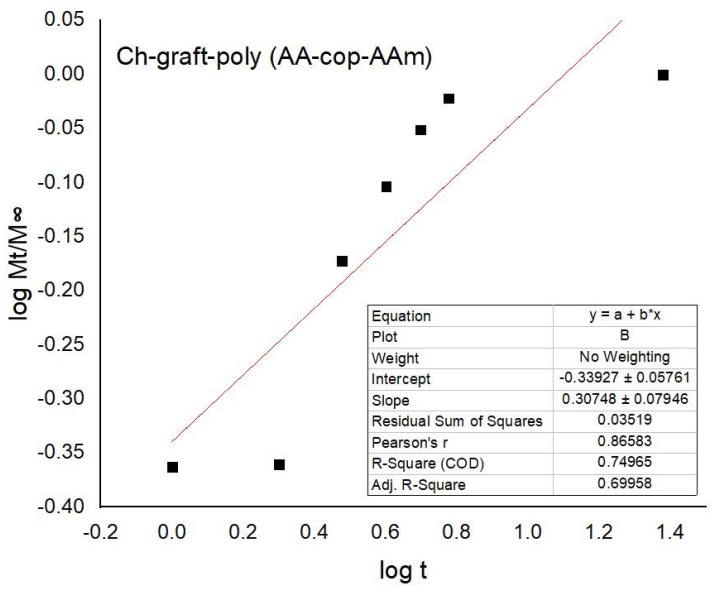
Drug release kinetics of the Ch-graft-poly (AA-cop-AAm) at pH 9.4.

The kinetic study of the DS drug diffusion from the samples was performed. By applying the Fick’s law to the percent drug release, the values of ’*n*’, ’*k*’ and ’*r*’ were assessed. As the value of ’*n*’ is below 0.5 for all the samples proves that drug discharge took place by Fickian diffusion. The ’*r*’ value approaching unity supports the linear release of the DS—as pictured in Figure 19 and presented in Table 3.

## 4. Conclusions

Chitosan was chemically modified by binary graft copolymerization of comonomers AAm and AN onto it by using free radical initiator KPS at optimal reaction conditions recorded for the grafting of AA (as unitary graft copolymerization was already reported). Morphological and chemical structural amendments after grafting were studied by characterization techniques and swelling study accomplished at pH 2.2,7.0,7.4 and 9.4 with respect to contact period 2–4 h. Binary graft copolymers swelled to much greater extent than chitosan due to numerous pores and more hydrophilicity and maximum $P_{s}$ was verified at pH 9.4.

To see future prospective of modified biopolymers, ternary graft copolymers, composites of biopolymers, composite graft copolymers can be synthesized with more functionality incorporated onto the biopolymers. In the case of environmental remediation techniques, these can be employed for competitive metal ions sorption or dye adsorption independent or competitive too. These can also be exploited for competitive drug adsorption studies as well. The literature survey revealed that materials based on chitosan and its derivates are widely implemented in numerous applications, such as bio sorbents, polymer fillers, electrochemical sensors, etc. Thus, they use the unique properties of chitosan and can provide multifunctional materials [23,40].

The metal ion sorption properties of the polymers depend upon a number of aspects such as swelling, porosity, nature/number of functional groups availability, hydrophilicity, monomer’s and comonomer’s nature, extent of graft copolymerization, crosslinking, etc. The incorporated functional groups show sorption of metal ions by chelation adsorption or by polymer-analogous reactions because of opening up of polymeric matrix and pores during swelling. The swelling behavior of chitosan is enhanced by graft copolymerization of different monomers with hydrophilic functionalities. The opening of the polymeric matrix after swelling resulted into the improved availability of hydrophilic functional groups merged by graft copolymerization to interact to positive charged metal ions that imparts a significant role in the amount and choice of metal ion uptake. Apart from all these factors, the metal ion’s ionic potential, charge on the ion, size of metal ion, hydration sphere of these metal ions also determines the sorption and retentions of metal ions on the polymeric adsorbents. Binary grafted copolymers showed preference for the metal ion uptake as Fe(II) > Pb(II). Maximum uptake of the Fe(II) ions i.e., >88% was recorded at pH 7.0 whereas the maximum uptake of Pb(II) ions >84% took place at pH 9.4.

The authors of this work were preparing chitosan-based hydrogels. The hydrogels are the materials, which undergo exceptionally high swelling in the aqueous medium. In addition, we are doing the modifications on the biopolymer chitosan, which is having outstanding properties such as non-toxicity, biocompatibility, zero-waste generation.

However, less porosity, less solubility and flaky structure are some hinderances for its efficient applicability as hydrogel. These shortcomings can be improved by chemical modifications such as graft copolymerization. The resultant products being highly porous showcase more efficient swelling. The swelling helps the matrix to open up and interact with the drug/metal ions well.

**Figure 20:**
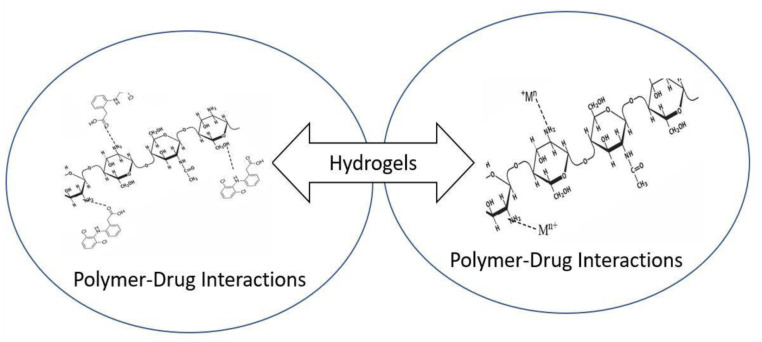
Chitosan interacting with drug DS as well as Metal ion.

The grafted sample proved comparatively high efficiency in biomedical field that is drug uptake/sustainable release as well as metal ions uptake. This is due to the factors such as increase in porosity (explained by SEM), increased amorphous nature (explained by XRD), More hydrophilic functional groups (explained by FTIR) and more thermal stability (explained by TGA/DTA studies). The interactions among polymers-drug and polymer-metal ions are electrostatic in nature. In Figure 20, the way how pristine chitosan is interacting with the drug and metal ions was presented.

In the present study we had chosen chitosan as a backbone due to its green properties such as non-toxic nature, hydrogel character, biodegradability, biocompatibility and most important zero-waste generation. It is chemically modified by free radical graft copolymerization of primary monomer AA, binary monomers AAm and AN. In the characterization techniques FTIR confirmed the grafting on to chitosan by the disappearance of O–H bending vibration peak of chitosan (indicating the mechanism of O–H side grafted reaction) and appearance of new peaks of the functional groups of monomers. The modifications were also confirmed by surface modification (more porosity supported by SEM images).

Discussing biomedical applications of novel stimuli-responsive binary graft copolymers as a potential candidate for precise release of DS, the polymeric samples swelled to the different extent at different pH. Therefore, drug molecules diffuse into (drug uptake) and diffuse out of (drug release) polymeric samples, which allow the possible use of dry or swollen polymeric samples as drug delivery vehicles. The results obtained showed that prolonged release of DS took place in alkaline media than neutral and acidic, which is again related with the swelling behaviour of the candidates. Hence, swelling with respect to pH as well as time will decide sustainability as well as targeted drug delivery. Drug uptake was highest for the Ch-graft-poly (AA-cop-AAm) (42.12%) and also showed best results for sustainable release within 24 h and targeted release also at the pH 9.4.

**Figure 21:**
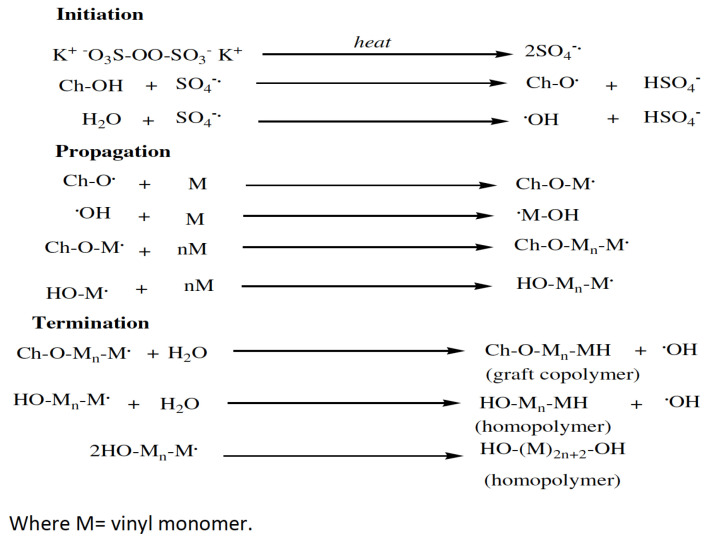
Mechanism of Graft copolymerization and crosslinking.

**Figure 22:**
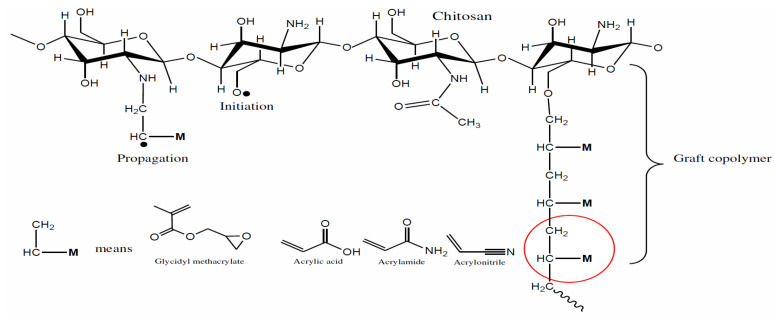
Binary Graft copolymerization.

In this study binary graft copolymerization was carried on chitosan, where primary monomer was acrylic acid and binary were acrylamide and acrylonitrile. The graft copolymerization was carried out by using KPS as a free radical initiator of AA and binary comonomers onto chitosan. KPS decomposes above 50 °C and undergoes homolytic cleavage to produce $SO_{4}^{-\cdot }$ radical anions. The radical anions abstract hydrogen atom from primary and secondary hydroxyl groups as well as from the amine groups of chitosan. However, the reactivity order of the chitosan functionality is primary −OH> secondary $–OH>-NH_{2}$ group. The mechanism of graft copolymerization is as follows (see: Figure 21 and Figure 22).

The preference for the formation of graft copolymer and homopolymers depends upon the reaction conditions such as the concentration of the monomer, the concentration of the initiator, the viscosity of the solvent, reaction temperature, etc.

This was also confirmed by FTIR studies, after grafting the product formation was confirmed by the disappearance of O–H bending vibration indicating the mechanism of O–H side grafted reaction.

In the future ternary graft copolymers can be synthesized with more functionality incorporated onto the biopolymers and these can be employed for competitive metal ions sorption as well as competitive drug adsorption. Based on author’s experience it is expected that chitin-based materials will be widely implemented in numerous applications, such as inter alia biosorbents, polymer fillers, and electrochemical sensors, thus, they use the unique properties of chitin and are able to provide a multifunctional materials [23,40].

Increased thermal stability supported by TGA/DTA studies, changes in the crystalinity confirmed by the Xray peak. There could be a concern about the evolution of chemical linkage of the monomer onto the backbone by the NMR. Based on the literature survey, in the current work the authors did not perform NMR studies, which is important in the polymer technology being an important analytical tool to understand derivatization, chemical linkage, etc.

Further research work should also include development of various reliable, highly sensitive, cost-effective based on inter alia chitosan biopolymers sensors, as these may have a very wide potential applicability in food industry and to become guarantee for food safety—in particular for issues related with infection/contamination of food. Bio-sensing can play a crucial role in that aspect [21,24,36,41]; it can also provide some kind of flexibility and lead to the rapid development of innovative, wearable sensors, medical devices or smart textiles. These electronic devices are built of flexible substrates—dominated by the traditional petro-materials-derived polymers, and active materials, including carbon materials, metals, metal oxides, semiconductors or conducting polymers; which are more expensive compared to the biosensors made of among the other chitin, and also less flexible and less efficient than natural biosensors [40,41,42,43,44,45,46]. A strong emphasis should also be put on biosensors’ development, in particular on those made of biopolymers (chitosan, cellulose and nanocrystalline cellulose), using which have shown promising results [47]. In addition, biopolymers are produced from renewable resources and therefore have been found as an interesting alternative to the traditional non-biodegradable materials due to their ability to get degraded by environmental agents. It is also important to mention that biopolymer-based sensors are less expensive and easier to make [25,41].

The current research work focused on providing solution to the environmental issues such as adulteration of the water bodies by the toxic pollutants such as heavy and toxic metal ions and providing an alternative in the biomedical field for the sustainable approach to drug delivery devices.

In this paper, the authors used biopolymer chitosan as a bio backbone. It has been modified by using vinyl monomers acrylic acid, acrylamide, and acrylonitrile to make binary graft copolymers through the free radical graft copolymerization technique by using KPS as a free radical initiator.

Optimum reactions conditions were evaluated by using the reaction scheme of variation of one parameter at a time and other parameters constant. iv. The structural, morphological, chemical, and thermal modifications in the chitosan before and after fabrication of binary grafted samples were analyzed by using characterization techniques XRD, SEM, TGA/DTA, FTIR, and swelling too in order to understand hydrogel character and stimuli responsiveness.

The potential of the hydrogels was explored in two different fields environment remediation and biomedical field.

In environment remediation removal of toxic bivalent metal ions Fe(II) and Pb(II) from the aqueous medium was performed by employing the prepared hydrogels as bio-sorbents. The grafting improved hydrogel characteristic of the polymers due to which Ch-graft-poly (AA-cop-AAm) showed 87.80% uptake of Pb(II) ions at pH 9.4 and temperature 25 °C and 88.40% and 88.00% adsorption of Fe(II) ions was shown by Ch-graft–poly (AA-cop-AAm) and the Ch-graft-poly (AA-cop-AN), respectively, at a similar temperature and pH 7.0 which is outstanding as compared to less than 50% uptake of Fe(II) and Pb(II) ions shown by bare chitosan. Therefore, the binary grafting resulted in the enhancement of adsorption properties.

In the biomedical applications model drug, the DS has loaded into the candidate biopolymers and its sustainable release with respect to time and pH was investigated. The results proved that the percent drug release was more controlled and quite sustainable at pH 7.4 and 9.4 by Ch-graft-poly (AA-cop-AAm) showing percent release of 96.87% and 97.65% in 6 h at pH 7.4 and 9.4, respectively.

Hence, the candidate polymeric material of chitosan proved to be excellent adsorbents to be used in industry, as well as in medical technology.

### Further Research Plans

As we are living in the polymer era, a lot of research is going on in this field. Earlier scientist explored all the possible polymers which out referring to their side impacts on environment as well as on flora and fauna. The polymers which are non-biodegradable or toxic will harm the environment in many ways. However, with the origin of green chemistry or with awareness about the environment conservation scientist are more interested in the exploration of biopolymers because of the zero- waste generation and bio-degradable nature of biopolymers.

The field of biopolymers is quite waste and there is lot to explore here, we can extract the biopolymers from the waste such as agro-waste (which is otherwise difficult to manage) and amend them according to the requirements by introducing desired functionalities through derivatization, composite formation, Chemical and radiation modifications too.

It is also possible toprepare nano polymers in order to explore the properties of these materials in nanoscale.

The biomaterials can be more exploited in biomedical field to extend the work with bioactivity detection and in vivo studies.

## Figures and Tables

**Table 1:** Thermogravimetric analysis.

Sr. No.	Polymeric Matrices				Thermo Gravimetric Data	DTG Peaks (°C)
		**Stages of**	**%**	**Left out**	**FDT**	**Exothermic**
		**Degradation**	**Weight Loss**	**Residue at 200 °C (%)**	**(°C)**	
1	Chitosan	26–100	12.44	86.01	576	70
		200–400	54.66			290
		400–576	31.35			570
4	Ch-graft-poly (AA-cop-AAm)	28.7–100	3.28	88.22	674	218
		200–400	43.64			377
		400–600	23.78			630
		600–674	18.6			
5	Ch-graft-poly (AA-cop-AN)	21.2–100	5.97	88.44	680	234
		200–400	31.56		389	
		400–500	13.85			654
		500–680	43.03			

**Table 2:** Drug uploading into the polymeric matrices with respect to time and pH.

Sr. No.	Polymer	Percent Drug Loaded	pH
1.	Chitosan	11.86	2.2
2.	Ch-graft-poly (AA-cop-AAm)	42.12	2.2
3.	Ch-graft-poly (AA-cop-AN)	5.56	2.2
4.	Chitosan	11.86	7.0
5.	Ch-graft-poly (AA-cop-AAm)	42.12	7.0
6.	Ch-graft-poly (AA-cop-AN)	5.56	7.0
7.	Chitosan	11.86	7.4
8.	Ch-graft-poly (AA-cop-AAm)	42.12	7.4
9.	Ch-graft-poly (AA-cop-AN)	5.56	7.4
10.	Chitosan	11.86	9.4
11.	Ch-graft-poly (AA-cop-AAm)	42.12	9.4
12.	Ch-graft-poly (AA-cop-AN)	5.56	9.4

Polymeric sample = 25 mg, Concentration of drug solution = 100 μg/mL.

**Table 3:** Drug Diffusion Kinetics.

Sr. No.	Polymeric Samples	9.4 pH		
		***n***	***K***	***r***
1	Chitosan	0.1795	0.6214	0.9948
2	Ch-graft-poly (AA-coo-AAm)	0.3011	0.5878	0.9954
3	Ch-graft-poly (AA-cop-AN)	0.5163	0.5246	0.9922

## Data Availability

Data available upon written request from the corresponding authors.

## Sample Availability

Samples available upon written request from the corresponding authors.

## Abbreviations

The following abbreviations are used in this manuscript:

AA	Acrylic acid
AAm	Acrylamide
AN	Acrylonitrile
KPS	Potassium persulfate
$H_{2}O$	Water
DS	Diclofenac sodium
SEM	Scanning Electron Microscopy
TGA	Thermogravimetric analysis
DTA	Differential thermal analysis
Ch	Chitosan
XRD	Xray Diffraction
FTIR	Fourier Transform Infrared Spectroscopy
pH	potential of hydrogen
IPN	Interpenetrating polymer network
semi−IPN	Semi-Interpenetrating polymer network
5−FU	Fluorouracil
NFD	nifedipine
HCl	Hydrochloride
NaOH	Sodium hydroxide
$KH_{2}PO_{4}$	Potassium dihydrogen phosphate
KCl	Potassium chloride
$Na_{2}B_{4}O_{7}.10H_{2}O$	sodium tetraborate decahydrate (borax)
$FeSO_{4}$	Ferrous sulfate
$Pb{\left(NO_{3}\right)}_{2}$	Lead (II) nitrate
DMF	Dimethylformamide
$P_{g}$	Percent grafting
%GE	Grafting efficiency
$P_{u}$	Metal ion percent uptake
$K_{d}$	Metal ion partition coefficient
$Q_{r}$	Metal ions retention capacity
$P_{du}$	Percent drug uptake
$D_{o}$	Total drug taken in solution
$D_{l}$	Drug left in supernatant liquid
$P_{dr}$	Percent drug release
$D_{s}$	Concentration of drug in solution
−graft−	Grafting
−cop−	copolymer
Ch−graft−poly (AA−cop−AAm)	Binary graft copolymer of comonomer acrylamide
Ch−graft−poly (AA−cop−AN)	Binary graft copolymer of comonomer acrylonitrile
LMW	Low molecular weight
